# Supplementary material for: Learning a genome-wide score of human–mouse conservation at the functional genomics level
Source: Nat Commun. 2021 May 3;12:2495. doi: 10.1038/s41467-021-22653-8 (PMC8093196; doi:10.1038/s41467-021-22653-8)
Supplement: Supplementary file 1 — Supplementary Information [file 41467_2021_22653_MOESM1_ESM.pdf]

Supplementary Figures

1. Effect of different weight ratios between positive and negative examples
2. Effect of ensembling and sampling training data on robustness
3. Effect of the number of ensembled neural networks on predictive power and robustness
4. Comparison of the LECIF score to scores learned with training data from either non-coding or coding regions
5. Effect of using fewer mouse functional genomic features
6. Overview of generating region-neighborhood LECIF score for pairs of human regions and extended mouse regions
7. Predictive power of region-neighborhood LECIF score for aligning pairs as a function of neighborhood size around each pair's mouse region
8. Predictive power of region-neighborhood LECIF score for aligning pairs binned by score percentile as a function of neighborhood size around each pair's mouse region
9. Distribution of mean LECIF score of peak calls provided to LECIF
10. Distribution of mean LECIF score in different mouse chromatin states
11. Distribution of LECIF score of GENCODE gene feature annotations
12. Cross-species similarity in chromatin states in pairs binned by LECIF score or human-only baseline score
13. Relative frequency of chromatin states in regions with low or high LECIF score
14. Chromatin states in low-scoring coding regions
15. LECIF score and human-only baseline score in topologically associated domain (TAD) boundaries
16. Scatter plot of the human-only baseline score and cross-species similarity in tissue-specific H3K27ac activity
17. Chromatin states in non-aligning pairs with high or low LECIF scores
18. Distribution of PhyloP score in aligning bases
19. Correlation between LECIF score and sequence constraint scores
20. Cross-species agreement in chromatin state frequency in pairs grouped based on LECIF score and PhyloP score
21. Relationship of LECIF score and PhyloP score in ConsHMM conservation states
22. Relationship of LECIF score and log-odds score for CpG island being classified as slowly evolving
23. Distribution of mean LECIF score of human genomic windows overlapping mouse insulin secretion QTL and human diabetes GWAS variant
24. A schematic of a pseudo-Siamese neural network

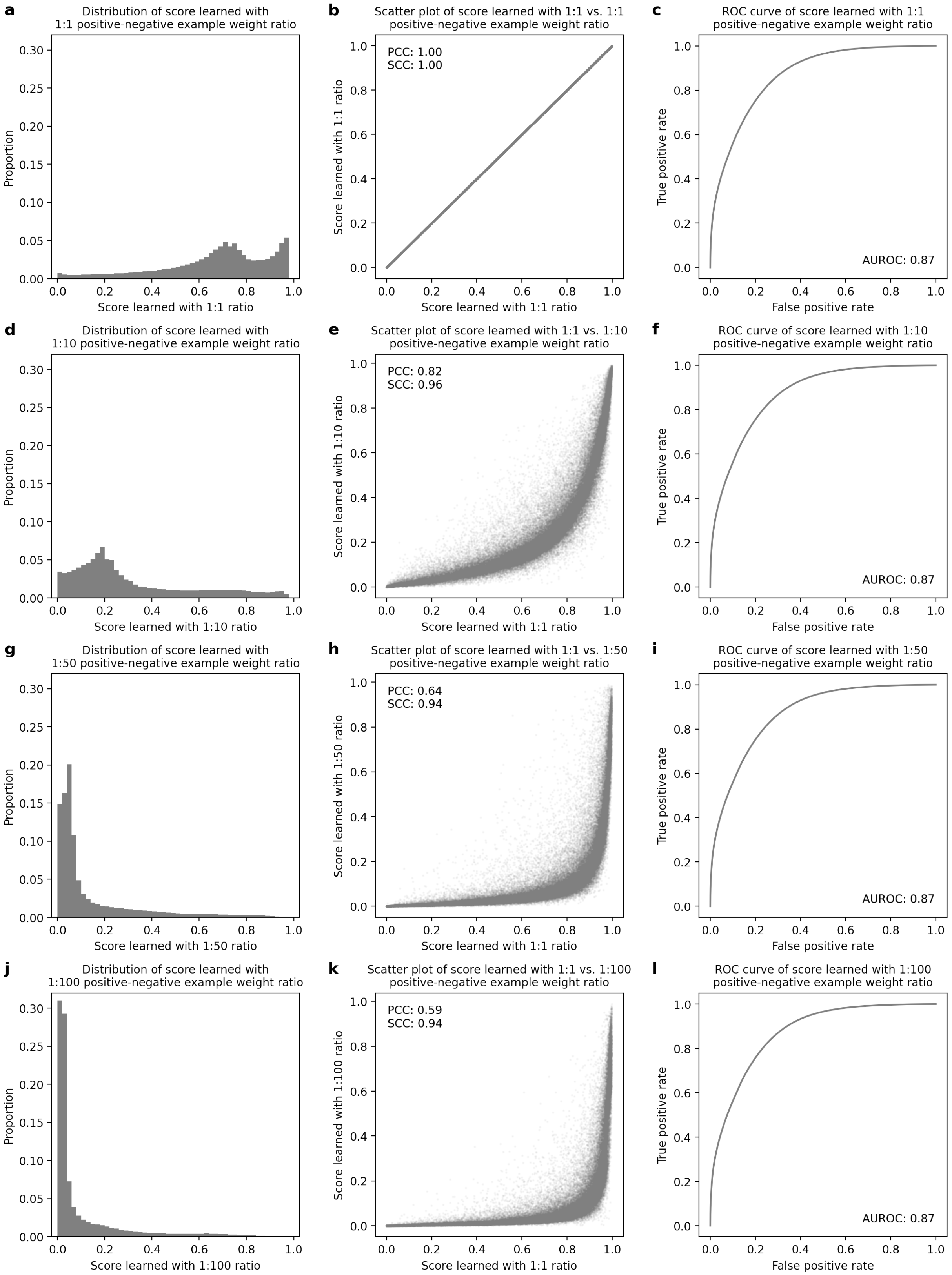
a
Distribution of score learned with 1:1 positive-negative example weight ratio
Proportion
Score learned with 1:1 ratio
b
Scatter plot of score learned with 1:1 vs. 1:1 positive-negative example weight ratio
PCC: 1.00
SCC: 1.00
Score learned with 1:1 ratio
Score learned with 1:1 ratio
c
ROC curve of score learned with 1:1 positive-negative example weight ratio
True positive rate
False positive rate
AUROC: 0.87
d
Distribution of score learned with 1:10 positive-negative example weight ratio
Proportion
Score learned with 1:10 ratio
e
Scatter plot of score learned with 1:1 vs. 1:10 positive-negative example weight ratio
PCC: 0.82
SCC: 0.96
Score learned with 1:10 ratio
Score learned with 1:1 ratio
f
ROC curve of score learned with 1:10 positive-negative example weight ratio
True positive rate
False positive rate
AUROC: 0.87
g
Distribution of score learned with 1:50 positive-negative example weight ratio
Proportion
Score learned with 1:50 ratio
h
Scatter plot of score learned with 1:1 vs. 1:50 positive-negative example weight ratio
PCC: 0.64
SCC: 0.94
Score learned with 1:50 ratio
Score learned with 1:1 ratio
i
ROC curve of score learned with 1:50 positive-negative example weight ratio
True positive rate
False positive rate
AUROC: 0.87
j
Distribution of score learned with 1:100 positive-negative example weight ratio
Proportion
Score learned with 1:100 ratio
k
Scatter plot of score learned with 1:1 vs. 1:100 positive-negative example weight ratio
PCC: 0.59
SCC: 0.94
Score learned with 1:100 ratio
Score learned with 1:1 ratio
l
ROC curve of score learned with 1:100 positive-negative example weight ratio
True positive rate
False positive rate
AUROC: 0.87

Supplementary Figure 1. Effect of different weight ratios between positive and negative examples. Comparisons of the LECIF score, which was learned with negative examples weighted 50 times more than positive examples, to alternative versions of the score learned with different weighting schemes. To generate each alternative version, we repeated the hyper-parameter search and prediction procedures with the same dataset, but with different weigthing scheme.

a,d,g,j. Distribution of a score learned with positive-negative example weight ratio of 1:1, 1:10, 1:50, and 1:100, respectively. Fifty equal-width bins were used to plot this histogram.

b,e,h,k. Scatter plot showing with a gray dot for each aligning pair of human and mouse regions a score learned with positive and negative examples weighted equally (x-axis) and a score learned with positive-negative example weight ratio of 1:1, 1:10, 1:50, and 1:100, respectively (y-axis). Pearson correlation coefficient (PCC) and Spearman correlation coefficient (SCC) between the two scores are shown in the top left. One hundred thousand pairs of human and mouse regions were randomly selected to be included in the scatter plot.

c,f,i,l. ROC curve of a score learned with positive-negative example weight ratio of 1:1, 1:10, 1:50, and 1:100, respectively, for differentiating positive and negative pairs. Mean ROC curve was obtained by classifying 100,000 positive and 100,000 negative examples randomly sampled with replacement from all available test examples 100 times Mean area under the ROC curve (AUROC) is shown in the bottom right corner. Standard deviation of the 100 AUROC values was under 0.001 for any weight ratio.

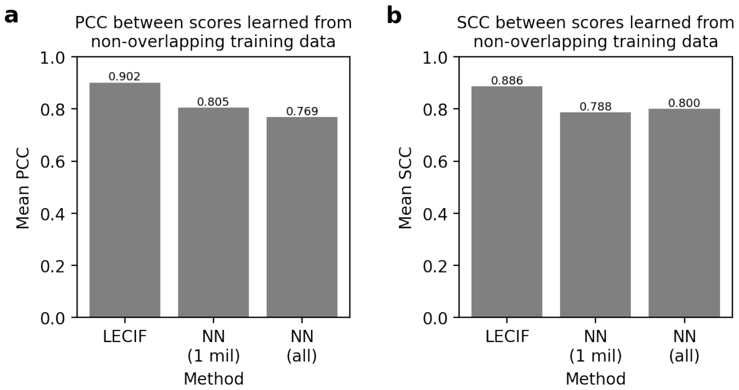

Supplementary Figure 2. Effect of ensembling and sampling training data on robustness. Analysis of the effect of the ensembling strategy of LECIF, which trains an ensemble of 100 neural networks, where each neural network (NN) is given 1 million positive and 1 million negative examples that are randomly sampled from all available training data, on the robustness of predictions. To evaluate the effect of ensembling, LECIF's robustness is compared to the average robustness of individual NN in the ensemble. Additionally, to evaluate the effect of sampling training data instead of using all available training data, LECIF's robustness is compared to the robustness of a single NN trained on all available training data (>2.2 million positive and >2.2 million negative examples). We measure the robustness by computing the **a.** PCC and **b.** SCC between scores generated by classifiers that were trained on non-overlapping set of chromosomes (**Methods**). 'NN (1 mil)' refers to the mean of 100 PCC or SCC computed from pairs of NN where the two NN are trained on different data. The NN were paired up randomly. 'NN (all)' refers to the PCC or SCC from two NN where each NN was trained on non-overlapping data, but without any down-sampling as done in 'NN (1 mil)'. The scores we compare here were generated for pairs of human and mouse regions held out from training, validation, and test (**Supplementary Data 2**). The same set of scores were used to compute both PCC and SCC. These results confirm that LECIF leads to more robust predictions than any individual NN in the ensemble or a NN trained on all available data.

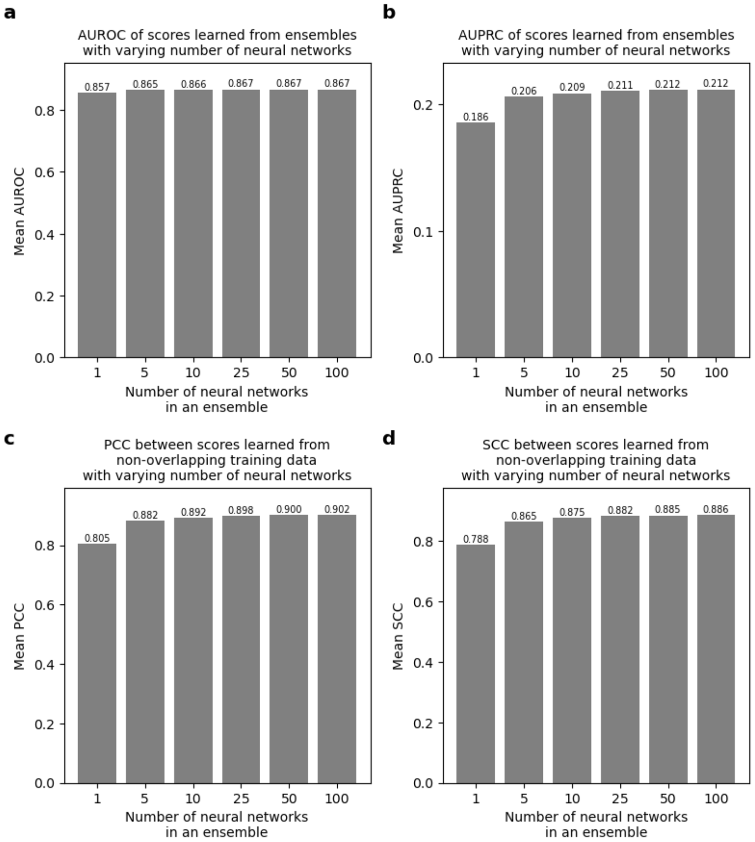

Supplementary Figure 3. Effect of the number of ensembled neural networks on predictive power and robustness.

Analysis of the effect of the number of neural networks used in LECIF, which trains an ensemble of 100 neural networks (NN), on classification performance and robustness of predictions. LECIF's predictive performance and robustness are compared to those of ensembles with fewer NN.

a. Effect of the number of NN in an ensemble on the area under the receiver operating characteristic curve (AUROC). Given 100 individual NN trained in LECIF, for each number of NN shown in the x-axis, *x*, we select at most 100 different ensembles, each of which is a combination of *x* NN. If there are 100 or fewer possible combinations, all are used. Otherwise, 100 combinations are randomly selected from all possible combinations. For each ensemble, we generated its prediction for test data by averaging the predictions from its NN. This test data was held out from training and validation of the NN. We finally computed AUROC for each ensemble and obtain the mean AUROC for each *x* by averaging the AUROCs over all ensembles consisting of *x* neural networks. Negative examples were weighted 50 times more than positive examples when computing AUROC.

b. Similar to **a** except showing area under the precision-recall curve (AUPRC) instead of AUROC. The same procedure and test data were used as **a**.

c. Similar to **a** except showing PCC between scores learned from different training data instead of AUROC. Ensembles were selected as done in **a** except we generated their predictions for held-out data that was excluded from all training, validation, and test (**Methods**). Given the ensembles generated for each number of NN shown in the x-axis, we computed PCC between scores predicted by two ensembles, each trained on non-overlapping training data. If there are multiple ensembles trained on different data, but with the same number of NN, then the two ensembles are matched randomly. We then computed the mean PCC for each number of NN by averaging the PCCs over the pairs of ensembles.

d. Similar to **c** except showing SCC instead of PCC.

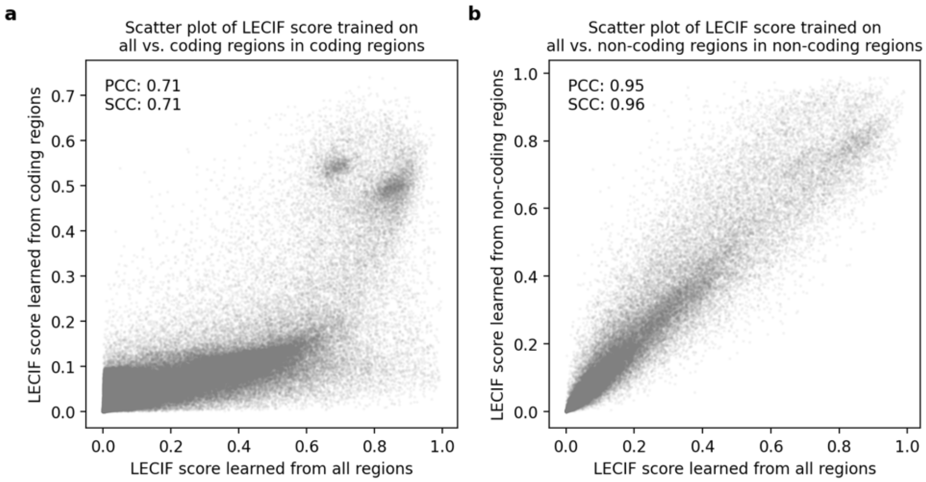

Supplementary Figure 4. Comparison of the LECIF score to scores learned with training data from either non-coding or coding regions.

To evaluate the effect of splitting training examples into coding and non-coding, we learned two separate scores, one from coding examples and the other from non-coding examples (**Methods**). A pair of human and mouse regions was considered coding if the human region overlapped any coding sequence and considered non-coding otherwise. The same procedure for learning the LECIF score was applied to learn a score from non-coding examples. The same procedure for learning the LECIF score was also done for coding examples, except, due to limited number of coding examples, all available training and tuning examples were used for hyperparameter search and then each classifier with optimized parameters was trained on 10,000 positive and 10,000 negative training examples. The scores learned separately on coding and non-coding regions are largely similar to the original LECIF score.

a. Scatter plot showing with a gray dot for a coding region its LECIF score learned from all regions (x-axis) and its score learned from coding regions (y-axis). Pearson correlation coefficient (PCC) and Spearman correlation coefficient (SCC) between the scores are shown in the top left. One hundred thousand pairs of human coding regions were randomly selected to be included in the scatter plot.

b. Similar to **a** except showing with a gray dot for a non-coding region its LECIF score learned from all regions (x-axis) and score learned from non-coding regions (y-axis).

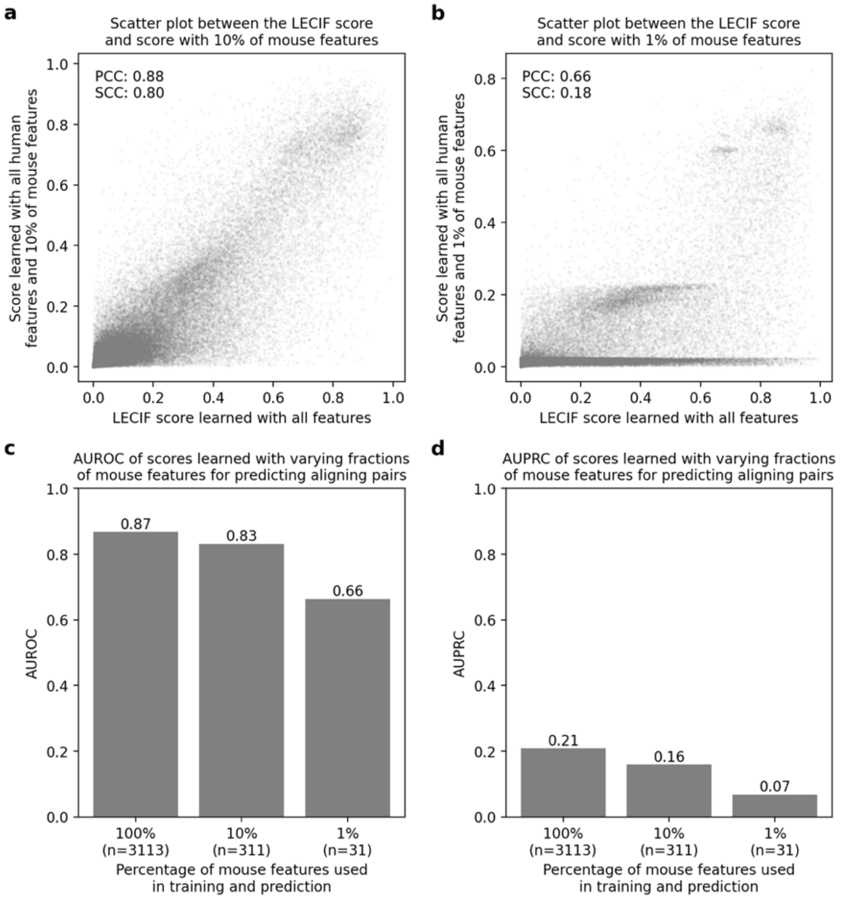

Supplementary Figure 5. Effect of using fewer mouse functional genomic features.
To examine the contribution of mouse data to LECIF, we learned two alternative scores using LECIF, one with 10% of the original mouse features and the other with 1% (**Methods**). Specifically, to sample 10% of the mouse features, we randomly selected 6 out of 66 epigenomes in the 15-state ChromHMM chromatin state annotations, selecting 90 chromatin state features. We then additionally sampled 221 features from those corresponding to mouse DNase-seq, ChIP-seq, RNA-seq, and CAGE experiments. To sample 1% of the mouse features, we randomly selected 31 features from those corresponding to mouse DNase-seq, ChIP-seq, RNA-seq, and CAGE experiments. Both scores were learned with all human features originally used in LECIF.

a. Scatter plot showing with a gray dot for each aligning pair of human and mouse regions the LECIF score learned with all features (x-axis) and the alternative score learned with 10% of mouse features. Pearson correlation coefficient (PCC) and Spearman correlation coefficient (SCC) between the two scores are shown in the top left. One hundred thousand pairs of human and mouse regions were randomly selected to be included in the scatter plot.

b. Similar to **a** except showing the alternative score learned with 1% of mouse features in the y-axis.

c. Bar plot showing mean AUROC of the LECIF score learned with all features and the alternative scores learned with all human features and 10% or 1% of mouse features for differentiating aligning pairs from randomly mismatched pairs. One hundred AUROCs were obtained by classifying 100,000 positive and 100,000 negative examples randomly sampled with replacement from all available test examples 100 times, as done in **Fig. 2c**. Mean AUROC is shown above each bar. Standard deviation of the 100 AUROC values was under 0.001 for all scores.

d. Similar to **c** except showing AUPRC instead of AUROC.

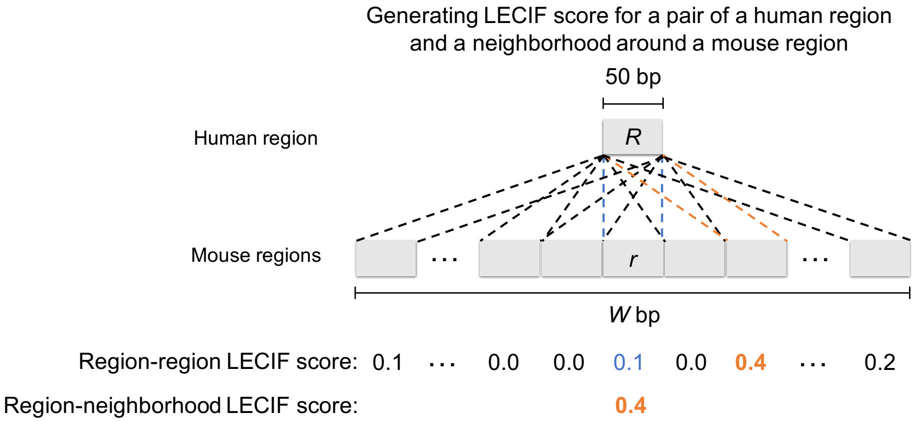

Supplementary Figure 6. Overview of generating region-neighborhood LECIF score for pairs of human regions and extended mouse regions.

Illustration of how LECIF is used to generate region-neighborhood LECIF score for a pair of a human region and a neighborhood of a mouse region. A given 50-bp human region *R* is compared to a set of multiple 50-bp mouse regions in a neighborhood of length *W* bp centered around a mouse region *r*. Each comparison (pair of dashed lines) results in a region-region LECIF score. For a pair of human region and a neighborhood in mouse, we define the region-neighborhood LECIF score as the maximum of all the region-region LECIF scores. In this example, the region-region LECIF score of the aligning human and mouse regions (blue; *R* and *r*) is 0.1. The maximum region-region LECIF score, 0.4, comes from the human region paired up with a mouse region near the aligning mouse region (orange). As a result, in this example, the region-neighborhood LECIF score is 0.4. We evaluated using the region-neighborhood LECIF score to predict aligning pairs, as an alternative to using the region-region LECIF score of the aligning human and mouse regions. Results of the evaluation are shown in **Supplementary Fig. 7**.

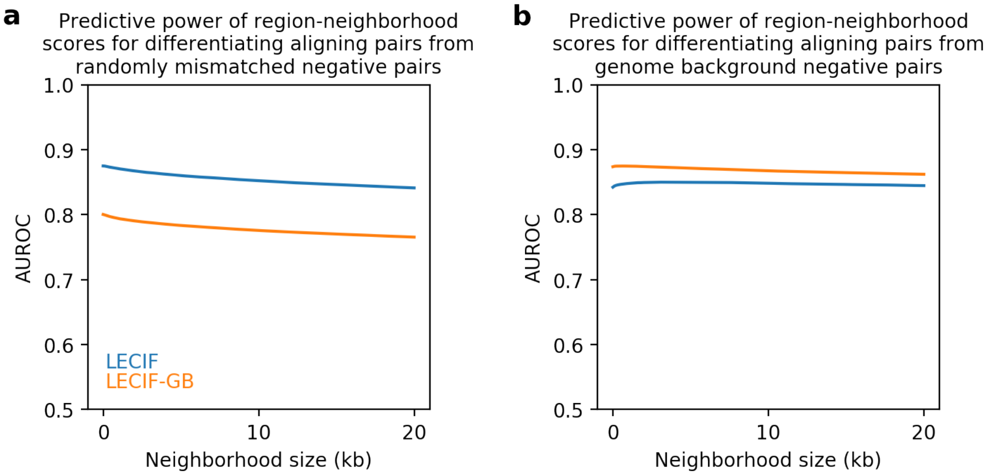

Supplementary Figure 7. Predictive power of region-neighborhood LECIF score for aligning pairs as a function of neighborhood size around each pair's mouse region.
We evaluate the predictive power of the region-neighborhood LECIF score of aligning human and mouse regions as a function of neighborhood size. We also evaluate using a LECIF-Genome Background (LECIF-GB) score in place of LECIF score in this analysis. LECIF-GB was trained with 'genome background' negative examples, which are pairs of human and mouse regions randomly selected from the entire human and mouse genomes (**Methods**). Shown for LECIF (blue) and LECIF-GB (orange) is the area under the ROC curve (AUROC) for differentiating positive examples from negative examples as a function of the size of the neighborhood centered around each pair's mouse region. Positive examples are pairs of human and mouse regions that align to each other. Negative examples are either **a.** randomly mismatched human and mouse regions that align somewhere in the other species (equivalent to the negative examples provided to LECIF) or **b.** genome background (equivalent to the negative examples provided to LECIF-GB). The neighborhood size varies from 0 to 20 kb with increments of 100 bp. Given a particular neighborhood size of W, the region-neighborhood score for each pair of human and mouse regions was the maximum region-region scores of any pair consisting of the human region and any mouse region within $0.5*W$ bp from the aligning mouse region of the pair (**Methods**; **Supplementary Fig. 6**). This region-neighborhood LECIF score was then used to predict aligning pairs. We note that a neighborhood size of 0 gives region-region LECIF and LECIF-GB scores. For each comparison, the same set of 100,000 positive and 100,000 negative test examples, which were on chromosomes excluded from training and validation, were used to compute the AUROC. In this analysis, there was no advantage in using the region-neighborhood LECIF score, as defined, compared to using the region-region LECIF score and similarly for LECIF-GB.

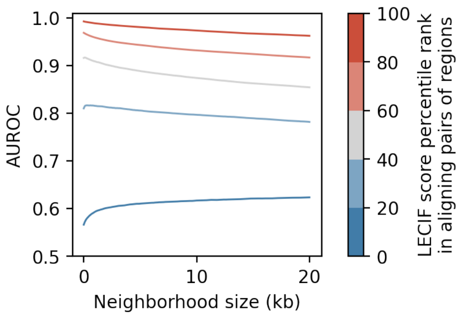

Supplementary Figure 8. Predictive power of region-neighborhood LECIF score for aligning pairs binned by score percentile as a function of neighborhood size around each pair's mouse region. Similar to **Supplementary Fig. 7a**, except we first bin aligning pairs into five bins based on their region-region LECIF score percentile rank at the aligning regions. For each bin, we evaluate the predictive power of the region-neighborhood LECIF score of aligning human and mouse regions as a function of neighborhood size. Each line corresponds to a percentile rank bin and is colored based on the color bar on the right. When measuring AUROC, for every positive example falling into a percentile rank bin, we provide a negative example that consists of the same human region of the positive example and a randomly chosen mouse region that aligns somewhere else in the human genome. While extending the neighborhood around each pair's mouse region does not improve predictive power in general, it does help when the aligning regions are scoring low and hard to distinguish from randomly mismatched pairs.

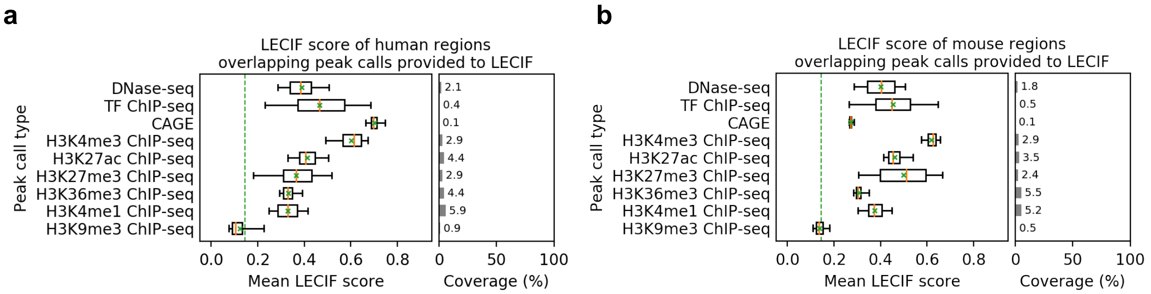

Supplementary Figure 9. Distribution of mean LECIF score of peak calls provided to LECIF.
a. Left panel shows for each type of functional genomic experiments listed the distribution of mean LECIF score over experiments of that type in human. The mean LECIF score for an experiment is computed based on averaging the LECIF score of regions overlapping a peak call from the experiment. The set of experiments are the same as provided to LECIF as input features. Each distribution is represented by a boxplot with median (orange solid line), mean (green 'x'), 25th and 75th percentiles (box), and 5th and 95th percentiles (whisker). Green dashed vertical line across the entire left panel denotes the genome-wide mean LECIF score. Right panel shows mean coverage of each type of peak call across all human regions that align to the mouse genome. Human regions in all aligning pairs of human and mouse regions (n=32,285,361) as defined in **Methods** were used to generate this plot. The number of experiments for each peak call type is reported in **Supplementary Data 1**. Source data are provided as a Source Data file.
b. Similar to **a** except for mouse experiments instead of human**.** Mouse regions in all aligning pairs of human and mouse regions (n=32,285,361) were used to generate this plot. Source data are provided as a Source Data file.

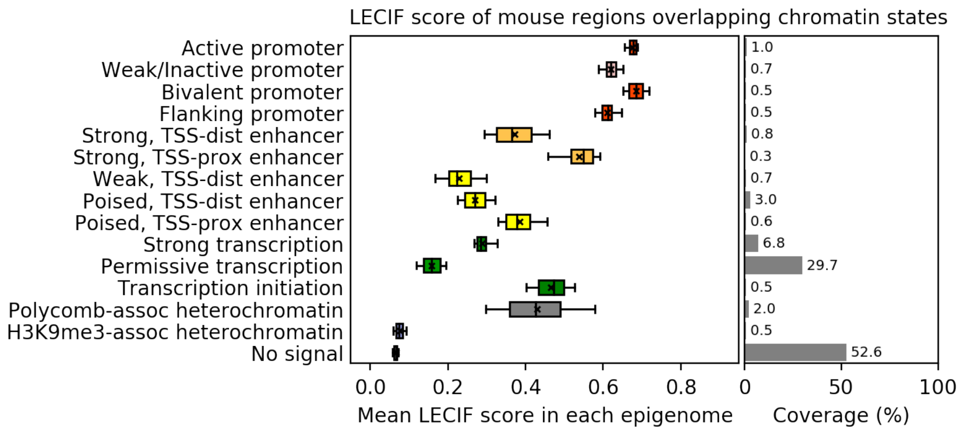

Supplementary Figure 10. Distribution of mean LECIF score in different mouse chromatin states. Similar to **Fig. 2e** except for mouse chromatin state annotations[1,2] instead of human**.** Left panel shows for each chromatin state from a model learned in mouse the distribution of mean LECIF score over different epigenomes (n=66). The mean LECIF score for a chromain state in an epigenome is computed by averaging the LECIF score of regions overlapping the chromatin state in the epigenome. Each distribution is represented by a boxplot with median (black vertical line), mean (black 'x'), 25th and 75th percentiles (box), and 5th and 95th percentiles (whisker). Right panel shows mean coverage of each state across mouse regions in all aligning pairs of human and mouse regions. Mouse regions in all aligning pairs of human and mouse regions (n=32,285,361) as defined in **Methods** were used to generate this plot. State colors were assigned to match the state colors of the 25-state human ChromHMM model[3] shown in **Fig. 2e** based on state descriptions. Source data are provided as a Source Data file.

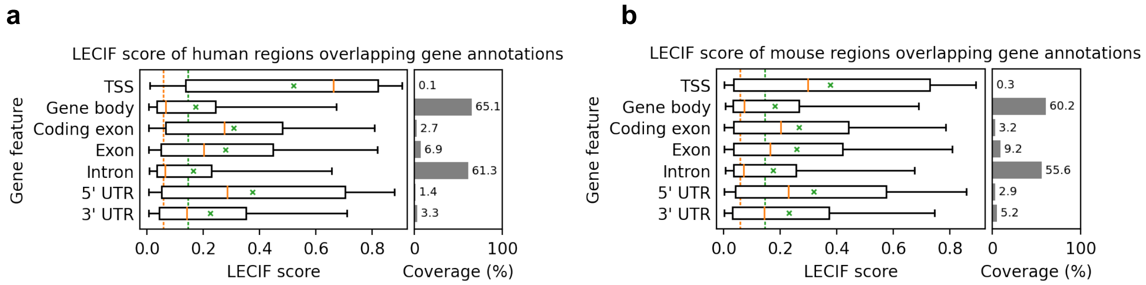

Supplementary Figure 11. Distribution of LECIF score of GENCODE gene feature annotations.
a. Left panel shows the distribution of LECIF score in human regions overlapping indicated GENCODE gene feature annotations. Each distribution is represented by a boxplot with median (orange solid line), mean (green 'x'), 25th and 75th percentiles (box), and 5th and 95th percentiles (whisker). Dashed vertical lines in orange and green across the entire left panel denote the genome-wide median and mean LECIF scores, respectively. Right panel shows coverage of each annotation across all human regions that align to the mouse genome. Human regions in all aligning pairs of human and mouse regions (n=32,285,361) were used to generate this plot. TSS: transcription start site; CDS: coding sequence; UTR: untranslated region.
b. Similar to **a** except for mouse regions overlapping mouse gene feature annotations instead of human. Mouse regions in all aligning pairs of human and mouse regions (n=32,285,361) as defined in **Methods** were used to generate this plot.

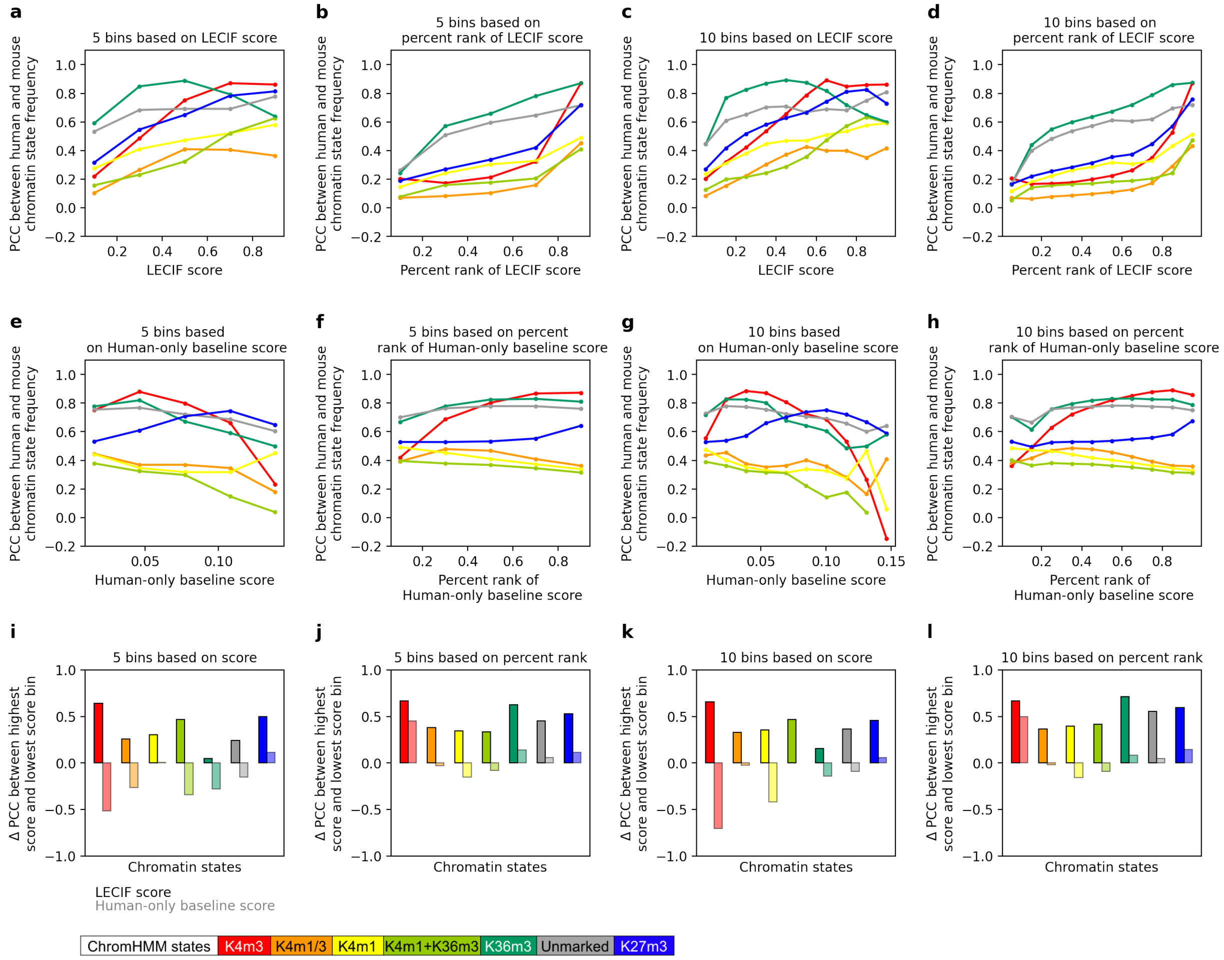

Supplementary Figure 12. Cross-species similarity in chromatin states in pairs binned by LECIF score or human-only baseline score.

Extended version of the analysis in **Fig 3b**. **a-d.** Cross-species agreement in chromatin state[2,4] frequency in pairs of aligning human and mouse regions binned by the LECIF score for a ChromHMM model learned jointly between human and mouse. Pairs are binned using **a.** 5 equal-width bins based on the LECIF score, **b.** 5 bins based on the percentile rank of the LECIF score, **c.** 10 equal-width bins based on the LECIF score, or **d.** 10 bins based on the percentile rank of the LECIF score. Binning based on the percentile rank results in similar number of pairs in each bin, whereas binning based on the score results in varying number of pairs in each bin. For each state and aligning region, we computed the frequency of the state across cell and tissue types for human and mouse separately. We then, for each state and bin, computed the PCC between the corresponding human and mouse frequencies for that state across all aligning pairs within the bin (**Methods**). The values are shown with colored circles according to the chromatin state legend on the bottom from Ref. [4]. The circles for the same state are connected with lines based on piecewise linear interpolation. **d** is identical to **Fig. 3b**. Source data are provided as a Source Data file.

e-h. Similar to **a-d**, respectively, except using the human-only baseline score instead of the LECIF score.

i-l. Shown for each chromatin state (x-axis) is the difference in the chromatin state's PCC between pairs from the highest score and lowest score bin Δ(PCC), based on either the LECIF score (bold-colored bars) or human-only baseline score (light-colored bars). Each panel corresponds to the two panels above it in the same column. The ΔPCC values are shown with colored bars according to the chromatin state legend

on the bottom from Ref. [4] and the score used for binning the pairs (bold for LECIF score, light for human-only baseline score).
This figure illustrates that pairs of human and mouse regions with high LECIF score show stronger cross-species agreement in chromatin state frequency than pairs with low LECIF score. It also highlights that pairs with high human-only baseline score do not consistently show stronger cross-species agreement than pairs with low human-only baseline score.

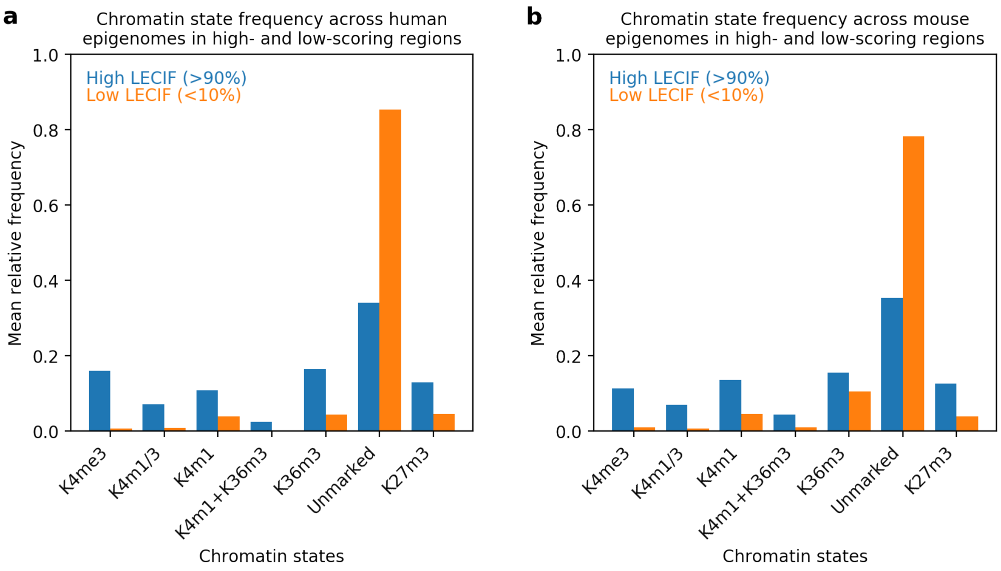

Supplementary Figure 13. Relative frequency of chromatin states in regions with low or high LECIF score.

Comparing relative frequency of chromatin states[2,4] for a seven state ChromHMM model learned jointly between human and mouse in high LECIF score (>90th percentile; blue) and low LECIF score (<10th percentile; orange) regions. The comparison is shown both for **a.** human and **b.** mouse regions. The chromatin states are the same as in **Fig. 3b** and **Supplementary Fig. 12**. For a species, the mean relative frequency of a chromatin state in a set of regions satisfying the LECIF score threshold was computed by averaging over epigenomes the fraction of those regions overlapping the chromatin state in each epigenome. These figures illustrate that regions with low LECIF score are more likely to be annotated with the 'Unmarked' chromatin state in both human and mouse than regions with high LECIF score.

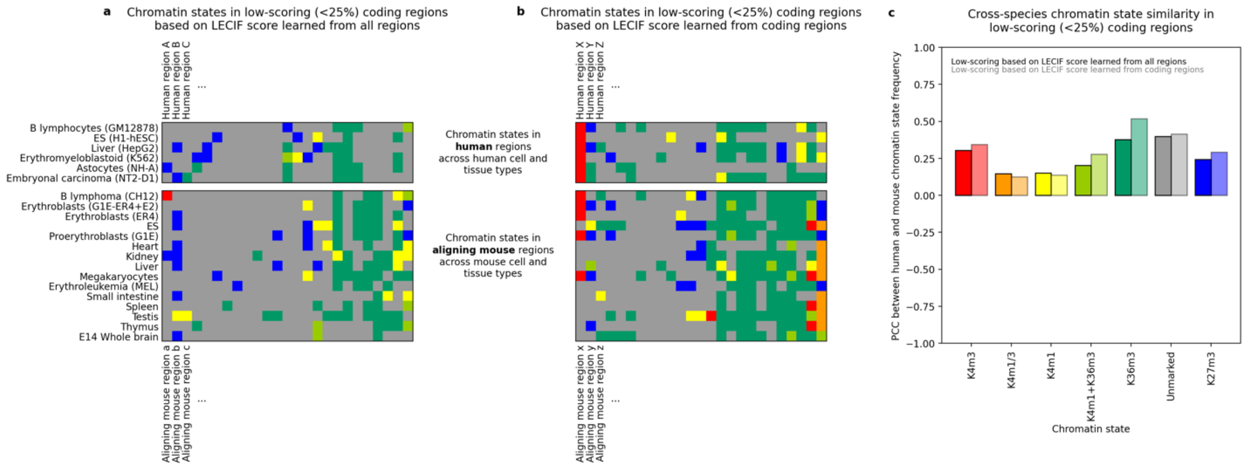

Supplementary Figure 14. Chromatin state similarity in low-scoring coding regions.

As described in **Supplementary Figure 4**, we learned an alternative score using pairs with coding human regions. Here we examine human and mouse chromatin states in low-scoring coding regions based on either the original LECIF score learned from all regions or the alternative score learned from coding regions. Coding regions that score low according to either scores exhibit weak cross-species similarity in their chromatin states as expected.

a. ChromHMM chromatin state[2,4] annotations in randomly selected pairs that include a human coding region with low LECIF score. The pairs were selected based on whether their human regions overlapped GENCODE annotation of coding sequence (CDS). Each row in the top sub-panel corresponds to a human cell or tissue type. Each row in the bottom sub-panel corresponds to a mouse cell or tissue type. Each column is a randomly selected pair with a human coding region with low LECIF score among all pairs with a human coding region (<25th percentile among coding regions). Each cell shows the color of the chromatin state with which the human or mouse region (column) is annotated in a specific cell or tissue type (row). The chromatin state model and state coloring are the same as in **Fig. 3b** and **Supplementary Fig. 12**. Pairs (columns) were ordered based on hierarchical clustering applied to their chromatin state annotations using Ward's linkage with optimal leaf ordering[5].

b. Same as **a**, but with pairs selected based on the alternative score learned from coding regions instead of the LECIF score.

c. Shown for each chromatin state (x-axis) is the state's PCC in low-scoring pairs with a human coding region based on the LECIF score (<25th percentile among coding regions; bold-colored bars) or the alternative score learned from coding training data (light-colored bars). Each state's PCC was computed as explained in **Fig. 3b** and **Supplementary Fig. 12** where the correlation is computed between the state's frequencies in human cell or tissue types and its frequencies in mouse cell or tissue types across all low-scoring pairs restricted to human coding regions.

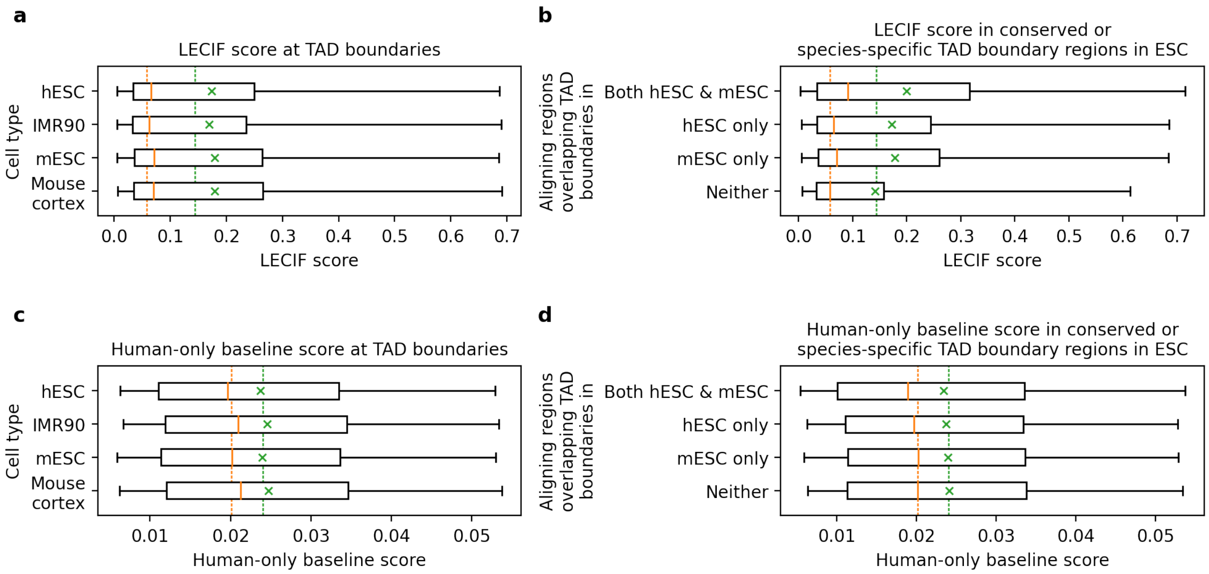

Supplementary Figure 15. LECIF score and human-only baseline score in topologically associated domain (TAD) boundaries[6].

a. Box plot showing the distribution of LECIF score of pairs with a human or mouse genomic region overlapping TAD boundaries in different human or mouse cell types. Top two cell types listed along the y-axis are human cell types, and the other two are mouse cell types. Each distribution is represented by a boxplot with median (orange solid line), mean (green 'x'), 25th and 75th percentiles (box), and 5th and 95th percentiles (whisker). Orange and green dashed lines vertical lines across the entire panel denote the genome-wide median and mean LECIF scores, respectively. There were 1,488,669, 1,344,362, 1,731,487, and 1,995,527 pairs of human and mouse regions as defined in **Methods** overlapping TAD boundaries in human embryonic stem cells (hESC), IMR90, mouse embryonic stem cells (mESC), and mouse cortex, respectively.

b. Similar to **a** but showing the distribution of LECIF score of pairs with human and mouse regions with respect to their overlap with TAD boundaries in embryonic stem cells (ESC). Top distribution corresponds to aligning human and mouse regions overlapping TAD boundaries in both hESC and mESC ('Both hESC & mESC'; n=82,075). Second and third distributions correspond to aligning pairs with either human or mouse region overlapping TAD boundaries in ESC ('hESC only' and 'mESC only'; n=1,406,056 and 1,234,447, respectively). Bottom distribution corresponds to the remaining pairs which are those with neither region overlapping TAD boundaries in ESC (n=29,552,172).

c-d. Similar to **a-b**, respectively, except for human-only baseline score instead of LECIF score.

These results show that LECIF score is higher at TAD boundaries than average, which are known to be highly conserved between human and mouse, and also higher in conserved TAD boundary regions than in species-specific TAD boundary regions. These patterns are not consistently observed with human-only baseline score.

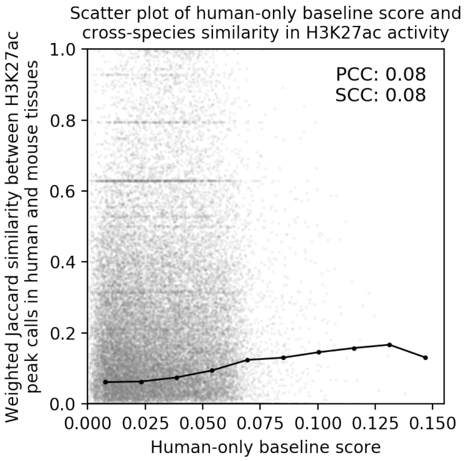

Supplementary Figure 16. **Scatter plot of the human-only baseline score and cross-species similarity in tissue-specific H3K27ac activity.**

Similar scatter plot to **Fig. 3a** except for the human-only baseline score (**Methods**) instead of the LECIF score. The scatter plot shows with a gray dot for each aligning pair of human and mouse regions the human-only baseline score (x-axis) and cross-species similarity of matched tissue-specific H3K27ac activity (y-axis). The H3K27ac activity for a region in a tissue and species is quantified as the fraction of experiments in the tissue type of the species with peak calls overlapping the region. The cross-species similarity of the tissue-specific H3K27ac activity is quantified as the weighted Jaccard similarity coefficient over 14 matched tissue types (**Methods**). PCC and SCC computed from all aligning pairs are shown in the top right. In black circles the mean similarity coefficient of pairs binned by the LECIF score with ten equal-width bins spanning from the minimum to maximum of the human-only baseline score is shown. These circles are connected with lines determined based on piecewise linear interpolation. One hundred thousand random aligning pairs were sampled to plot the scatter plot. This analysis shows that the human-only baseline score exhibits weaker agreement with cross-species similarity in tissue-specific H3K27ac activity compared to the LECIF score (PCC: 0.08 vs 0.45 and SCC: 0.08 vs 0.42).

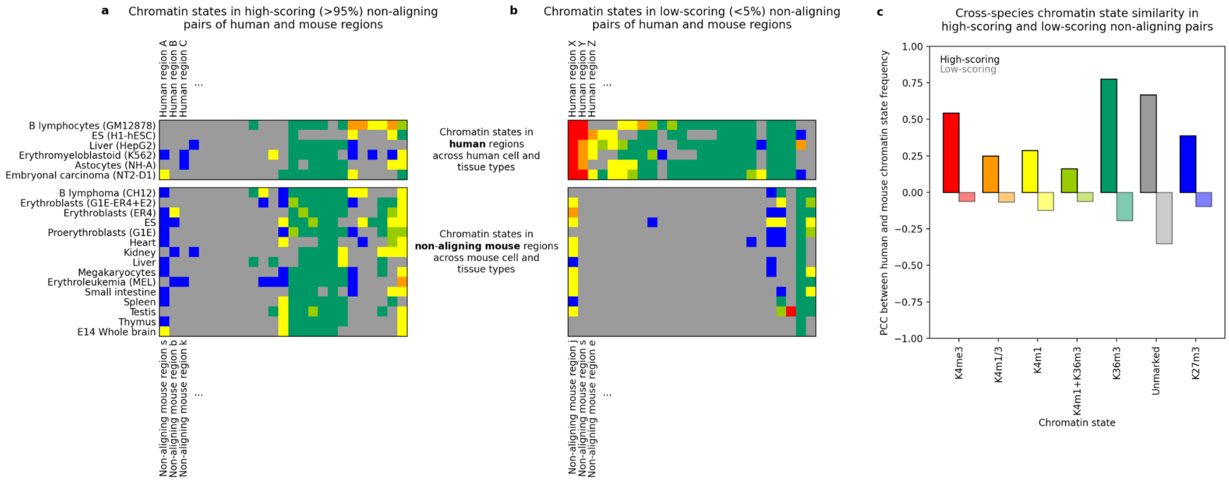

Supplementary Figure 17. Chromatin states in non-aligning pairs with high or low LECIF scores.
a. ChromHMM chromatin state[2,4] annotations in randomly selected pairs of non-aligning human and mouse regions with high LECIF score. The pairs were selected from negative test examples which consist of randomly mismatched pairs of human and mouse regions that do not align to each other (**Methods**). All human and mouse regions included in these pairs do align somewhere in the other species. Each row in the top sub-panel corresponds to a human cell or tissue type. Each row in the bottom sub-panel corresponds to a mouse cell or tissue type. Each column is a randomly selected non-aligning pair with high LECIF score among all non-aligning pairs (>95th percentile). Each cell shows the color of the chromatin state with which the human or mouse region (column) is annotated in a specific cell or tissue type (row). The chromatin state model and state coloring are the same as in **Fig. 3b** and **Supplementary Fig. 12**. Pairs (columns) were ordered based on hierarchical clustering applied to their chromatin state annotations using Ward's linkage with optimal leaf ordering[5].
b. Same as **a**, but with randomly selected non-aligning pairs with low LECIF score (<5th percentile).
c. Shown for each chromatin state (x-axis) is the state's PCC in non-aligning pairs with high (>95th percentile; bold-colored bars) or low (<5th percentile; light-colored bars) LECIF score. Each state's PCC was computed as explained in **Fig. 3b** and **Supplementary Fig. 12** where the correlation is computed between the state's frequencies in human cell or tissue types and its frequencies in mouse cell or tissue types across 100,000 pairs with either high or low LECIF scores. Pairs were randomly sampled from negative test examples as done in **a** and **b**.

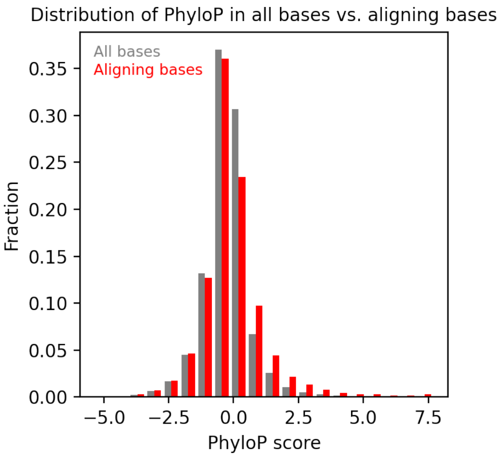

Supplementary Figure 18. **Distribution of PhyloP score in aligning bases.**
Comparison of the distribution of PhyloP score (100 vertebrate) in human genomic bases in general (gray) and bases that align to mouse (red). 1 million bases annotated by PhyloP score were randomly sampled from the genome. Shown in gray is the distribution of PhyloP score of all 1 million bases. Shown in red is the distribution of PhyloP score of bases that align to mouse among the 1 million bases. Twenty equal-width bins ranging from -5 to 8 were used to plot the histogram, covering more than 99% of the score distribution. Bins outside the range are not shown. This comparison demonstrates that although aligning bases have a slightly higher distribution of sequence constraint than all bases they still have a wide distribution of constraint.

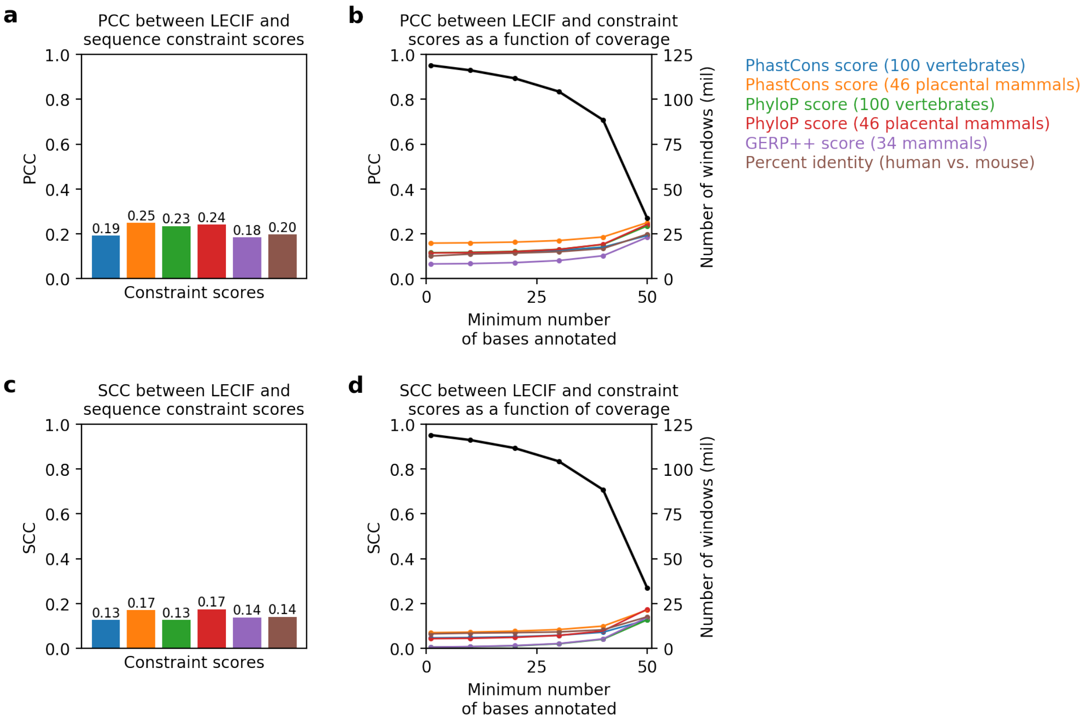

Supplementary Figure 19. Correlation between LECIF score and sequence constraint scores.
a. Shown for a set of sequence constraint scores[7–9] is the PCC computed between the LECIF score and a constraint score. For a given constraint score, to compute the PCC, we first slid a non-overlapping 50-bp genomic window across the human genome and selected windows with all 50 bases annotated by both the LECIF score and the given constraint score. We then computed the mean LECIF score and mean constraint score for each selected window. The PCC for a constraint score is the PCC between those two sets of values. Each resulting PCC is shown with a bar colored according to the legend on the right. Percent identity is defined as the number base-pairs with matching nucleotides (e.g. G in human and G is mouse) within a given window divided by 50. Source data are provided as a Source Data file.
b. PCC between the LECIF score and constraint scores as a function of the minimum number of bases required to be annotated in the genomic windows. Also shown is the number of windows selected to compute the PCC. The PCC for a constraint score is computed as described in **a,** except windows with at least *n* bases annotated by the LECIF score and the constraint score of interest are selected, where *n* varies from 1 to 50. The two scores being compared need not annotate the same set of bases in each window. The PCC are shown with colored circles according to the y-axis on the left and legend in the top right. The circles for the same constraint score are connected with lines based on piecewise linear interpolation. The rightmost values where the minimum number of bases annotated equals 50 correspond to the PCC shown in **a**. Black circles show the number of windows in millions that had at least *n* bases (x-axis) annotated by the LECIF score and constraint scores according to the y-axis on the right. These circles are connected with lines based on piecewise linear interpolation. All six comparisons of the LECIF score to constraint scores had the same number of selected genomic windows. Source data are provided as a Source Data file.
c-d. Similar to **a-b**, respectively, except for SCC instead of PCC.
These results show that the LECIF score is moderately correlated with sequence constraint scores, and that the correlations are weaker as we include windows with fewer bases annotated by the scores within each genomic window.

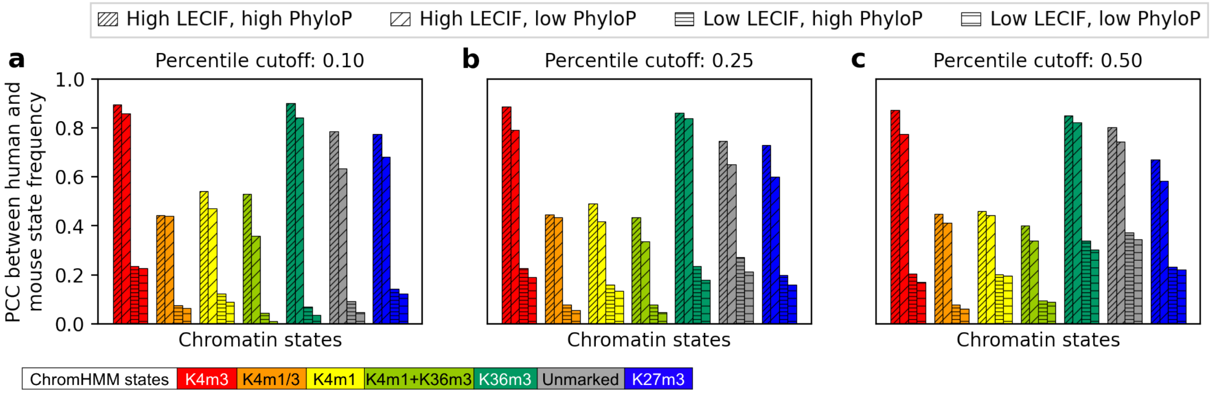

Supplementary Figure 20. Cross-species agreement in chromatin state frequency in pairs grouped based on LECIF score and PhyloP score.

a. ChromHMM chromatin state[4,10] frequency correlation between human and mouse in pairs of aligning human and mouse regions grouped based on whether their LECIF score and human PhyloP score[7] (defined based on a 100-way vertebrate alignment) were high (>90th percentile) or low (<10th percentile). The chromatin states are the same as in **Fig. 3b** and **Supplementary Fig. 12**. Separate bars are shown for each combination of high or low score of LECIF or PhyloP as indicated based on the legend at top. For the low PhyloP case, we required that there be a low (<10th percentile) score at all annotated bases within 500 bp to ensure the low score was not driven by the higher resolution at which sequence conservation is defined. The frequency correlation for each state and a set of aligning pairs is quantified as the PCC between the human and mouse frequencies for that state across the pairs, as done in **Fig. 3b** and **Supplementary Fig. 12** (**Methods**). Any region that did not have a PhyloP score for all bases was discarded from this analysis. Bars for each state are colored according to the bottom legend, as previously defined in Ref. [4]. Source data are provided as a Source Data file.

b. Similar to **a** except using a percentile cutoff of 0.25 instead of 0.05. Scores above the 75th percentile are considered high, and scores below the 25th percentile are considered low.

c. Similar to **a** except using a percentile cutoff of 0.50 instead of 0.05. Scores above the median are considered high, and scores below the median are considered low.

These results demonstrate that pairs with high LECIF score exhibit strong cross-species agreement in chromatin state frequency even when there is a low PhyloP score in the region. In contrast, pairs with a high PhyloP score and a low LECIF score did not exhibit strong correlations.

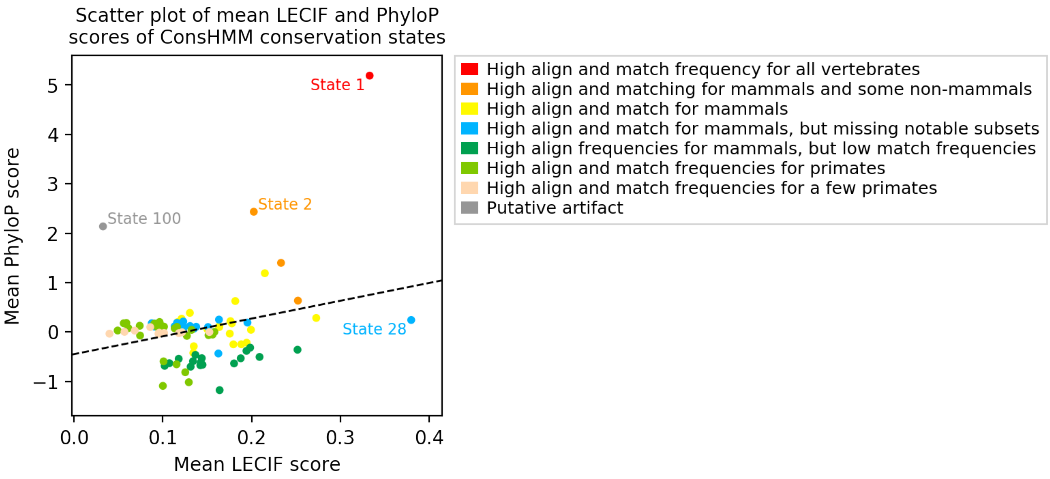

Supplementary Figure 21. Relationship of LECIF score and PhyloP score in ConsHMM conservation states.

We use a ConsHMM 100-conservation-state annotation of the human genome based on a 100-way vertebrate sequence alignment[11] to understand the relationship between the LECIF score and sequence constraint scores. The scatter plot shows with a dot for each ConsHMM conservation state the mean LECIF score (x-axis) and mean human PhyloP score[7] (y-axis; defined based on a 100-way vertebrate alignment). For each conservation state, the mean LECIF or PhyloP score is computed by averaging the score of bases overlapping the conservation state. Each dot is colored according to the eight major groups of conservation states listed in the legend on the right, as previously defined in Ref. [11]. Dashed line is a linear regression fit applied to the 100 data points. We label four noteworthy conservation states. State 28 (blue), which is the promoter enriched state, has the highest mean LECIF score and the 12th highest mean PhyloP score. State 1 (red), which is the most enriched state for exons, has the 2nd highest mean LECIF score and the highest mean PhyloP score. State 2 (orange), which is the state most enriched for enhancer chromatin states, has the 8th highest mean LECIF score and the 2nd highest mean PhyloP score. State 100 (gray), which is characterized by pseudogenes and putative artifacts in the multi-species sequence alignment[11], has the lowest mean LECIF score, while having the 3rd highest mean PhyloP score. Source data are provided as a Source Data file.

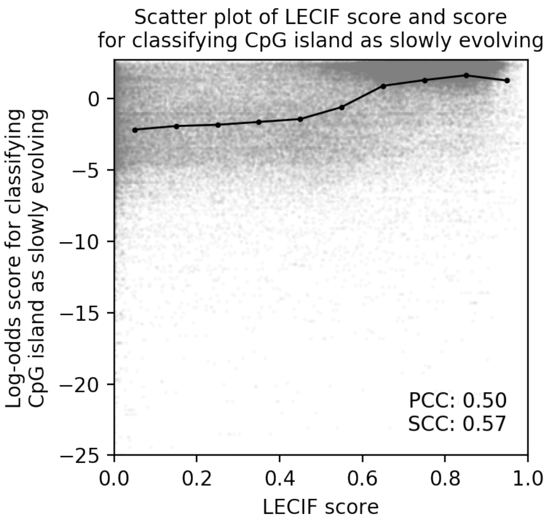

Supplementary Figure 22. Relationship of LECIF score and log-odds score for CpG island being classified as slowly evolving.

Scatter plot showing with a gray dot for each human CpG island the mean LECIF score (x-axis) and the log-odds score for classifying the CpG island as slowly evolving as opposed to quickly evolving (y-axis) from a previous study on primate CpG island sequence evolution[12]. In black circles the mean log-odds score for CpG islands binned by the LECIF score with ten equal-width bins is shown. These circles are connected with lines based on piecewise linear interpolation. One hundred thousand random human CpG islands annotated with the LECIF score were sampled to plot this scatter plot. PCC and SCC computed between the two scores across all CpG islands annotated with the LECIF score are shown in the bottom right. This illustrates that the LECIF score is positively correlated with the likelihood of a human CpG island being classified as slowly evolving as opposed to quickly evolving. Source data are provided as a Source Data file.

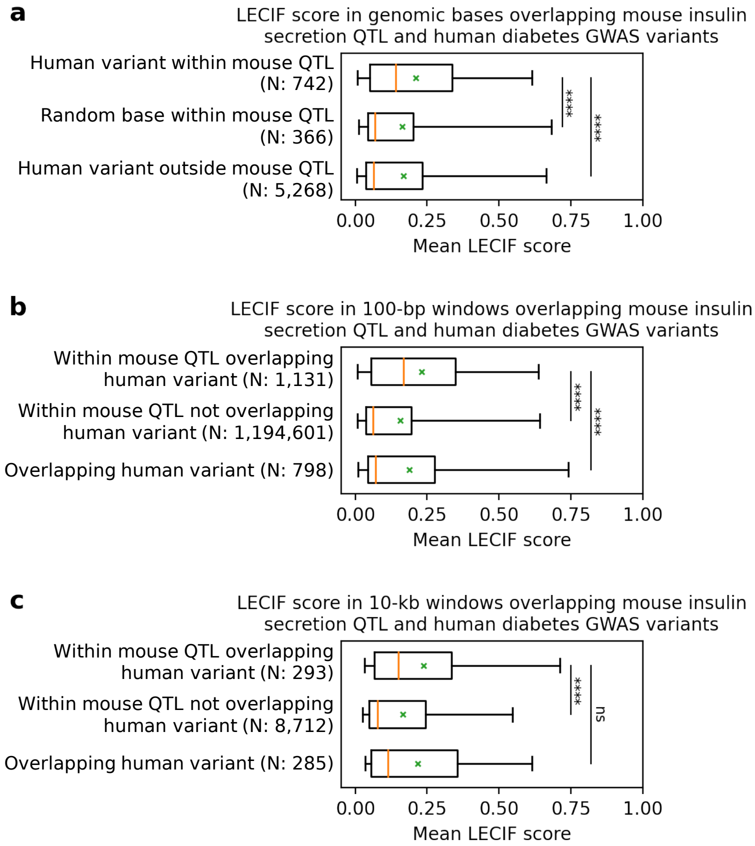

Supplementary Figure 23. Distribution of mean LECIF score of human genomic windows overlapping mouse insulin secretion QTL and human diabetes GWAS variant.

a. Distribution of mean LECIF score in genomic bases identified as human diabetes GWAS variant or overlapping a mapped mouse insulin secretion QTL or both[15]. The top group refers to human GWAS variants that lie within the mouse QTL mapped to human. The middle groups refers to random genomic bases that overlap the mapped mouse QTL where the bases were obtained by randomly permutating the locations of the human diabetes GWAS variants. The bottom groups refers to human GWAS variants that do not overlap any mapped mouse QTL. Displayed after each label is the number of bases corresponding to that group. Each distribution is represented by a boxplot with median (orange solid line), mean (green ‘x’), 25th and 75th percentiles (box), and 5th and 95th percentiles (whisker). **** denotes p-value below 0.0001 based on a two-sided Mann-Whitney U test. Specifically, the p-values for comparing the top vs. middle groups and top vs. bottom groups were 2e-6 and 1e-15, respectively. Source data are provided as a Source Data file.

b. Similar to **Fig. 6a**, but showing the distribution of mean LECIF score in non-overlapping 100-bp genomic windows, instead of 1-kb windows, identified as containing a human diabetes GWAS variant or overlapping a mapped mouse insulin secretion QTL or both[15]. The top group refers to windows that lie within the mouse QTL mapped to human and overlap the human GWAS variant. The middle group refers to windows within the mouse QTL that do not overlap the human GWAS variant. The bottom group refers to windows from the human genome that overlap the GWAS variant where the windows lie in loci obtained by randomly permuting the locations of the mapped mouse QTL. Displayed after each label is the number of windows corresponding to that group. Each distribution is represented by a boxplot with median (orange solid line), mean (green ‘x’), 25th and 75th percentiles (box), and 5th and 95th percentiles (whisker). All windows were obtained by sliding a fixed window across the QTL, and any window with less than half of the bases annotated with the LECIF score was excluded. **** denotes p-value below

0.0001, and ns denotes p-value above 0.05 based on a two-sided Mann-Whitney U test. Specifically, the p-values for comparing the top vs. middle groups and the top vs. bottom groups were 4e-54 and 7e-11, respectively. Source data are provided as a Source Data file.

c. Similar to **b**, but showing the distribution of mean LECIF score in non-overlapping 10-kb genomic windows, instead of 100-bp genomic windows. The p-values for comparing the top vs. middle groups and top vs. bottom groups were 5e-11 and 0.10, respectively. Source data are provided as a Source Data file.

a shows that human diabetes GWAS variants that overlap mouse insulin secretion QTL tend to have a higher LECIF score than the GWAS variants outside of the mouse QTL or bases that are not GWAS variants, but within the mouse QTL. **b** and **c** show the result of **Fig. 6a,** that human genomic windows that overlap both mouse insulin secretion QTL and human diabetes GWAS variant tend to have a higher LECIF score than windows that overlap only one of them and that this result also holds for other window sizes.

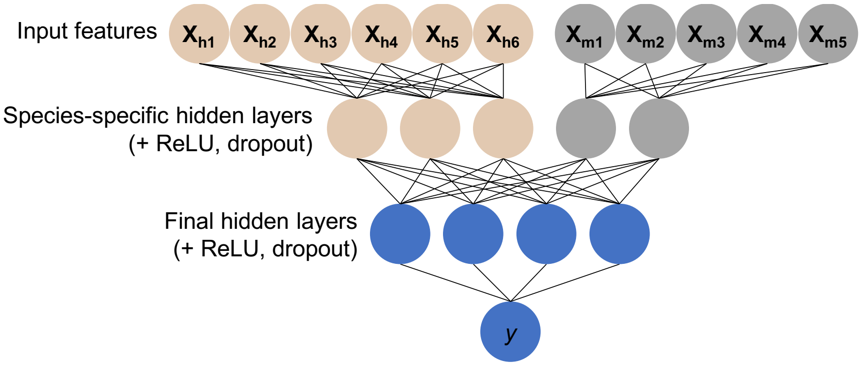

Supplementary Figure 24. A schematic of a pseudo-Siamese neural network.
A pseudo-Siamese neural network consists of two distinct sub-networks that do not share any weights[16]. The sub-network on the left (beige) takes in human feature vectors, $\mathbf{X_h}$, and the sub-network on the right (gray) takes in mouse feature vectors, $\mathbf{X_m}$. Each feature vector consists of multiple features, denoted as $\mathbf{X}_{\mathbf{h}i}$ or $\mathbf{X}_{\mathbf{m}i}$, with i ranging from 1 to total number of features. A final network (blue) takes in concatenated output vectors from the two sub-networks and generates the final prediction, y. Each layer within a sub-network is followed by a rectified linear unit (ReLU) and dropout is used in the training[17]. We only show a small number of input features, layers, and neurons here. **Supplementary Data 3** lists the hyper-parameters that define this architecture.

References

1. Gorkin, D. U. *et al.* An atlas of dynamic chromatin landscapes in mouse fetal development. *Nature* **583**, 744–751 (2020).
2. Ernst, J. & Kellis, M. ChromHMM: automating chromatin-state discovery and characterization. *Nat. Methods* **9**, 215–216 (2012).
3. Roadmap Epigenomics Consortium *et al.* Integrative analysis of 111 reference human epigenomes. *Nature* **518**, 317 (2015).
4. Yue, F. *et al.* A comparative encyclopedia of DNA elements in the mouse genome. *Nature* **515**, 355–364 (2014).
5. Bar-Joseph, Z., Gifford, D. K. & Jaakkola, T. S. Fast optimal leaf ordering for hierarchical clustering . *Bioinformatics* **17**, S22–S29 (2001).
6. Dixon, J. R. *et al.* Topological Domains in Mammalian Genomes Identified by Analysis of Chromatin Interactions. *Nature* **485**, 376–380 (2012).
7. Pollard, K. S., Hubisz, M. J., Rosenbloom, K. R. & Siepel, A. Detection of nonneutral substitution rates on mammalian phylogenies. *Genome Res.* **20**, 110–121 (2010).
8. Siepel, A. *et al.* Evolutionarily conserved elements in vertebrate, insect, worm, and yeast genomes. *Genome Res* **15**, (2005).
9. Davydov, E. V *et al.* Identifying a High Fraction of the Human Genome to be under Selective Constraint Using GERP++. *PLOS Comput. Biol.* **6**, e1001025 (2010).
10. Ernst, J. *et al.* Mapping and analysis of chromatin state dynamics in nine human cell types. *Nature* **473**, 43–49 (2011).
11. Arneson, A. & Ernst, J. Systematic discovery of conservation states for single-nucleotide annotation of the human genome. *Commun. Biol.* **2**, 248 (2019).
12. Cohen, N. M., Kenigsberg, E. & Tanay, A. Primate CpG Islands Are Maintained by Heterogeneous Evolutionary Regimes Involving Minimal Selection. *Cell* **145**, 773–786 (2011).
13. Finucane, H. K. *et al.* Partitioning heritability by functional annotation using genome-wide association summary statistics. *Nat. Genet.* **47**, 1228 (2015).
14. Gazal, S. *et al.* Linkage disequilibrium–dependent architecture of human complex traits shows action of negative selection. *Nat. Genet.* **49**, 1421 (2017).
15. Keller, M. P. *et al.* Gene loci associated with insulin secretion in islets from nondiabetic mice. *J. Clin. Invest.* **129**, 4419–4432 (2019).
16. Hughes, L. H., Schmitt, M., Mou, L., Wang, Y. & Zhu, X. X. Identifying Corresponding Patches in SAR and Optical Images With a Pseudo-Siamese CNN. *IEEE Geosci. Remote Sens. Lett.* **15**, 784–788 (2018).
17. LeCun, Y., Bengio, Y. & Hinton, G. Deep learning. *Nature* **521**, 436–444 (2015).
