# Supplementary material for: Learning a genome-wide score of human–mouse conservation at the functional genomics level
Source: Nat Commun. 2021 May 3;12:2495. doi: 10.1038/s41467-021-22653-8 (PMC8093196; doi:10.1038/s41467-021-22653-8)
Supplement: Supplementary file 3 — Description of Additional Supplementary Files [file 41467_2021_22653_MOESM3_ESM.pdf]

Description of Additional Supplementary Files

File Name: Supplementary Data 1

Description:

a. Summary of input features provided to LECIF. The first column contains the data type of features. The second and third columns contain the number of features generated from human and mouse experiments, respectively, of the data type specified in the first column. For ChromHMM chromatin state annotations, the total number of features generated from all chromatin state annotations after one-hot encoding, the number of epigenomes, and the number of states are shown. The fourth and last columns list the consortia from which the data type specified in the first column was collected. The last row contains the total number of human and mouse features.

b. Metadata for human DNase-seq and ChIP-seq experiments. The first column contains the index in the human feature vector X_h corresponding to each experiment. The second column contains the name of the bed file we downloaded which reports the peak calls from each experiment. The third and fourth columns contain the name of the epigenome and tissue group, respectively, in which each experiment was performed. Tissue groups were manually assigned to the experiments largely based on available metadata provided by the Roadmap Epigenomics Project and ENCODE but with some curation by the authors to allow grouping of experiments from different consortia and species for downstream analyses. The fifth column contains the type of assay performed (DNase-seq or ChIP-seq). The sixth column contains the assay target (left empty for DNase-seq experiments). The seventh column contains the consortia (Roadmap or ENCODE) that provided the data for each experiment . The last column contains the link to the bed file.

c. Metadata for human ChromHMM chromatin state annotations. The first and second columns contain the first and last indices of the 25 consecutive indices in the human feature vector X_h corresponding to the 25 states in each chromatin state annotation. The third column contains the name of the bed file containing each annotation. The last column contains the link to the bed file.

d. Metadata for human Cap Analysis Gene Expression (CAGE) experiments. A file containing a matrix of read counts in called peaks from all human CAGE experiments was downloaded from: http://fantom.gsc.riken.jp/5/datafiles/latest/extra/CAGE_peaks/hg19.cage_peak_phase1and2combined_co unts.osc.txt.gz. In this matrix, each row corresponds to a genomic interval and each column corresponds to a CAGE experiment. In the presented table, the first column contains the index in the human feature vector X_h corresponding to each CAGE experiment. The second column contains the name of the column in the peak call read count table that corresponds to each experiment. The third column contains the description of the cell type and conditions of each experiment.

e. Metadata for human RNA-seq experiments. The first column contains the index in the human feature vector X_h corresponding to each experiment. The second column contains the name of the bigWig file we downloaded which reports the genome-wide signal from each experiment. The third column contains the name of the epigenome in which each experiment was performed. The last column contains the link to the bigWig file.

f. Metadata for mouse DNase-seq and ChIP-seq experiments. Similar to b, except for mouse.

g. Metadata for mouse ChromHMM chromatin state annotations. Similar to c, except for mouse.

h. Metadata for mouse CAGE experiments. Similar to d, except for mouse. The peak call read count matrix for mouse was downloaded from: http://fantom.gsc.riken.jp/5/datafiles/reprocessed/mm10_latest/extra/CAGE_peaks_expression/mm10_fai r+new_CAGE_peaks_phase1and2_counts.osc.txt.gz

i. Metadata for mouse RNA-seq experiments. Similar to e, except for mouse.

File Name: Supplementary Data 2
Description:
a. Groups of pairs used for training classifiers that generate predictions for human regions in even chromosomes. The first column contains the purpose of each group of pairs defined in the next two columns and the total number of pairs in each group. Each group (first column) consists of pairs of aligning human and mouse regions from a specific set of chromosomes specified in the second and third columns, respectively.
b. Groups of pairs used for training classifiers that generate predictions for human regions in odd chromosomes. Similar to a, except for predictions in odd chromosomes in human.

File Name: Supplementary Data 3
Description: Hyper-parameter values considered and chosen for all methods. The first column contains each type of classifier trained in this study. The second column contains hyper-parameters tuned for each type of classifier. The third column contains the candidate values tried during hyper-parameter tuning. The following twenty columns contain chosen values for each hyper-parameter given a specific training data set or setting of the classifier specified in the first column. For the number of neurons in each layer in ENN and EFCNN, "n/a" is shown if the layer does not exist, as specified by another parameter (i.e. number of layers). The last two columns contain fixed parameters, if they exist, and the values to which they were fixed.
