# Supplementary material for: Learning a genome-wide score of human–mouse conservation at the functional genomics level
Source: Nat Commun. 2021 May 3;12:2495. doi: 10.1038/s41467-021-22653-8 (PMC8093196; doi:10.1038/s41467-021-22653-8)
Supplement: Supplementary file 7 — Reporting Summary [file 41467_2021_22653_MOESM7_ESM.pdf]

nature research

Corresponding author(s): Jason Ernst

Last updated by author(s): Mar 10, 2021

# Reporting Summary

Nature Research wishes to improve the reproducibility of the work that we publish. This form provides structure for consistency and transparency in reporting. For further information on Nature Research policies, see our Editorial Policies and the Editorial Policy Checklist.

## Statistics

For all statistical analyses, confirm that the following items are present in the figure legend, table legend, main text, or Methods section.

| n/a | Confirmed | |
|---|---|---|
| ☐ | ☒ | The exact sample size (*n*) for each experimental group/condition, given as a discrete number and unit of measurement |
| ☒ | ☐ | A statement on whether measurements were taken from distinct samples or whether the same sample was measured repeatedly |
| ☐ | ☒ | The statistical test(s) used AND whether they are one- or two-sided<br>*Only common tests should be described solely by name; describe more complex techniques in the Methods section.* |
| ☒ | ☐ | A description of all covariates tested |
| ☐ | ☒ | A description of any assumptions or corrections, such as tests of normality and adjustment for multiple comparisons |
| ☒ | ☐ | A full description of the statistical parameters including central tendency (e.g. means) or other basic estimates (e.g. regression coefficient) AND variation (e.g. standard deviation) or associated estimates of uncertainty (e.g. confidence intervals) |
| ☐ | ☒ | For null hypothesis testing, the test statistic (e.g. *F*, *t*, *r*) with confidence intervals, effect sizes, degrees of freedom and *P* value noted<br>*Give P values as exact values whenever suitable.* |
| ☒ | ☐ | For Bayesian analysis, information on the choice of priors and Markov chain Monte Carlo settings |
| ☒ | ☐ | For hierarchical and complex designs, identification of the appropriate level for tests and full reporting of outcomes |
| ☐ | ☒ | Estimates of effect sizes (e.g. Cohen's *d*, Pearson's *r*), indicating how they were calculated |

*Our web collection on statistics for biologists contains articles on many of the points above.*

## Software and code

Policy information about availability of computer code

| | |
|---|---|
| Data collection | No software used |
| Data analysis | Custom code: https://github.com/ernstlab/LECIF<br>Scikit-learn (version 0.19.1), Pyrcca (https://github.com/gallantlab/pyrcca) |

For manuscripts utilizing custom algorithms or software that are central to the research but not yet described in published literature, software must be made available to editors and reviewers. We strongly encourage code deposition in a community repository (e.g. GitHub). See the Nature Research guidelines for submitting code & software for further information.

## Data

Policy information about availability of data

All manuscripts must include a data availability statement. This statement should provide the following information, where applicable:

- Accession codes, unique identifiers, or web links for publicly available datasets
- A list of figures that have associated raw data
- A description of any restrictions on data availability

The human-mouse LECIF score is available at https://github.com/ernstlab/LECIF. Links to data files used to generate input features to LECIF are listed in Supplementary Data 1. Source data behind Figure 2e, 3ab, 4-6 and Supplementary Figures 9, 10, 12, 16, 19-23 are provided with this paper. The human-mouse pairwise alignment is available at http://hgdownload.cse.ucsc.edu/goldenpath/hg19/vsMm10/axtNet/. For TSS, gene body, intron, exon, coding exon, 5' UTR, and 3' UTR annotations, we used GENCODE annotations V31lift37 for human and VM23 for mouse. We downloaded these annotations along with classification of evolutionary dynamics of CpG islands35 and common SNPs (dbSNP v7)37 from the UCSC Table Browser45. The HGMD variants that we used were variants annotated as 'regulatory mutations' in the April 2012 public release of HGMD database36,55. The following URLs contain data sets that were used in the heritability partitioning analysis: Baseline annotation set41: https://storage.googleapis.com/broad-alkesgroup-public/LDSCORE/1000G_Phase3_baselineLD_v2.1_ldscores.tgz;

Age at menarche56: https://www.reprogen.org; Body mass index, height57: http://www.broadinstitute.org/collaboration/giant/index.php/GIANT_consortium_data_files; Coronary artery disease58: http://www.cardiogramplusc4d.org/data-downloads; Education attainment59: https://www.thessgac.org/data; HDL cholesterol level, LDL cholesterol level, triglyceride level60: http://csg.sph.umich.edu/willer/public/lipids2010; Rheumatoid arthritis61: http://plaza.umin.ac.jp/yokada/datasource/software.htm; Schizophrenia62, smoking63: www.med.unc.edu/pgc/downloads; Type 2 diabetes64: http://www.diagram-consortium.org/downloads.html.

# Field-specific reporting

Please select the one below that is the best fit for your research. If you are not sure, read the appropriate sections before making your selection.

- [x] Life sciences
- [ ] Behavioural & social sciences
- [ ] Ecological, evolutionary & environmental sciences

For a reference copy of the document with all sections, see nature.com/documents/nr-reporting-summary-flat.pdf

# Life sciences study design

All studies must disclose on these points even when the disclosure is negative.

| | |
|---|---|
| Sample size | n/a. No experimental data collected in this study. |
| Data exclusions | n/a. No experimental data collected in this study. |
| Replication | n/a. No experimental data collected in this study. |
| Randomization | n/a. No experimental data collected in this study. |
| Blinding | n/a. No experimental data collected in this study. |

# Reporting for specific materials, systems and methods

We require information from authors about some types of materials, experimental systems and methods used in many studies. Here, indicate whether each material, system or method listed is relevant to your study. If you are not sure if a list item applies to your research, read the appropriate section before selecting a response.

## Materials & experimental systems

| n/a | Involved in the study | |
|---|---|---|
| x | | Antibodies |
| x | | Eukaryotic cell lines |
| x | | Palaeontology and archaeology |
| x | | Animals and other organisms |
| x | | Human research participants |
| x | | Clinical data |
| x | | Dual use research of concern |

## Methods

| n/a | Involved in the study | |
|---|---|---|
| x | | ChIP-seq |
| x | | Flow cytometry |
| x | | MRI-based neuroimaging |
